# Prospective Associations of Serum Vitamin B12, Homocysteine, and Ferritin Levels with Probable Sarcopenia

**DOI:** 10.3390/nu18091362

**Published:** 2026-04-25

**Authors:** Inkyung Baik

**Affiliations:** 1 Department of Biopharmaceutical Chemistry, College of Science and Technology, Kookmin University, Seoul 02707, Republic of Korea; ibaik@kookmin.ac.kr; Tel.: +82-2-910-4774; 2 Department of Pharmaceutical Engineering, College of Science and Technology, Kookmin University, Seoul 02707, Republic of Korea

**Keywords:** vitamin B12, folic acid, homocysteine, ferritins, muscle strength, sarcopenia, prospective studies

## Abstract

**Background/Objectives**: Previous cross-sectional studies investigated the associations of low handgrip strength (HS), a primary indicator of probable sarcopenia (PS), with biomarkers related to anemia. However, existing evidence is inconsistent, and data establishing causality remain limited. The present prospective study aimed to evaluate whether serum vitamin B12, folate, homocysteine (Hcy), and ferritin levels are associated with PS risk. **Methods**: This study analyzed data from 1930 adults aged 45–76 years who had normal muscle quantity at baseline. Serum biomarkers were assessed at baseline and PS defined by low HS was determined at 6-year follow-up. The modified Poisson regression method was employed to calculate multivariable risk ratios (RRs) and 95% confidence intervals (CIs). **Results**: Among all participants, PS risk was inversely related to serum vitamin B12 levels (*p* = 0.06), while it was lowest in the high-normal ranges of serum Hcy (12.1–15 μmol/L) and ferritin (101–200 ng/mL) levels. The RRs (95% CIs) for PS risk were 0.73 (0.60, 0.89) and 0.75 (0.64, 0.87) for high-normal Hcy and ferritin categories, respectively, compared with the lowest category. On examining the associations of elevated Hcy and ferritin levels with PS risk, age was identified as a significant modifier for elevated Hcy levels (>15 μmol/L) (*p* for interaction < 0.05); a reduced risk was observed in younger participants, whereas an increased risk was noted in older participants. **Conclusions**: These findings suggest that high-normal ferritin levels may be optimal for alleviating PS risk, irrespective of age, and that elevated Hcy levels could be detrimental for older adults in preventing PS risk.

## 1. Introduction

Muscle strength is considered an essential characteristic in the definition of sarcopenia, and ongoing discussions aim to establish an international consensus on this matter [1,2,3]. Handgrip strength (HS) has been widely utilized as a convenient and cost-effective tool for assessing muscle strength and diagnosing sarcopenia; especially, the sole measurement of HS is considered a practical tool to define probable sarcopenia (PS) [2]. A recent systematic literature review has summarized the factors associated with HS [4]. Previous epidemiological studies have identified demographic, socioeconomic, psychological, and lifestyle factors, as well as medical conditions and biomarkers that correlate with HS. However, investigations into the relation between nutritional biomarkers and HS are limited [4].

There are some epidemiological data on the associations of plasma vitamin B12, folate, or homocysteine (Hcy) levels with muscle strength and physical performance [5,6,7,8,9]. Findings concerning plasma Hcy levels, which are influenced by vitamin B12 and folate status, have produced inconsistent results. Notably, conflicting sex-specific results have been reported; certain studies have observed a significant association between elevated Hcy levels and low HS exclusively in men [5] or in women [6,7] while others have reported no such association in women [8,9]. Hcy, a sulfur-containing, non-essential amino acid, plays a role in the re-methylation pathway, where it is converted into the essential amino acid methionine by methionine synthase, with vitamin B12 serving as a cofactor and folate as a methyl donor. Consequently, deficiencies in either of these vitamins potentially disrupt the re-methylation pathway, resulting in elevated Hcy levels [10]. Nevertheless, few studies have examined both vitamins to establish a direct link between Hcy levels and HS [6].

Evidence linking low Hb levels or iron deficiency anemia to low HS or sarcopenia is coherent [11]. Nonetheless, the reciprocal nature of this association renders the causal relationship unclear [12]. To elucidate the impact of iron status on HS, further investigations should employ more sensitive markers than Hb for the early detection of iron deficiency. Recent studies have utilized ferritin or transferrin saturation as indicators of iron status [13,14]. One study reported a positive association between these markers and HS [13], while another suggested that elevated ferritin levels, indicative of iron overload, may pose a risk factor for sarcopenia, defined in terms of HS, muscle function, and mass [14]. Considering that inflammation potentially elevates ferritin levels [15], controlling potential confounding factors related to inflammatory conditions is imperative when analyzing the association between ferritin levels and HS.

Consequently, elevated levels of high Hcy and ferritin have recently raised concerns, as these overload states may adversely affect muscle health through increased oxidative stress and inflammation while also reflecting underlying medical conditions that contribute to sarcopenia [7,14]. For the general population, the optimal levels of Hcy and ferritin associated with low HS, as well as the detrimental levels linked to it, remain a topic of debate.

Therefore, the current prospective study aimed to evaluate the associations of serum vitamin B12, folate, Hcy, and ferritin levels, including overload states of Hcy and ferritin, with the risk of PS, as defined by low HS for Asian adults. Specifically, this study sought to analyze sex- and age-specific associations while accounting for inflammatory states and other related biomarkers. Additionally, interactions with well-established risk factors, such as age, exercise, and protein intake, were assessed.

## 2. Participants and Methods

### 2.1. Study Design and Participants

This investigation utilized data from ‘the Ansung cohort’ study with prospective design, which is part of the Korean Genome and Epidemiology Study (KoGES). The KoGES datasets are available through the Korea National Institute of Health (KNIH) website (https://biobank.nih.go.kr) upon official request from researchers. The population and methodology of this cohort study have been detailed in previous reports [16,17]. Briefly, the Ansung cohort study enrolled middle-aged and older adult residents of Ansung city (Gyeonggi Province, Republic of Korea). To enroll cohort members, a two-stage cluster sampling method was utilized based on the information of a governing district from the telephone directory and demographic data from the 2000 Census. Eligible participants, who were men and women aged 40 years or older residing in Ansung city, were identified by telephone contact and invited to visit the Ajou University Medical Center for a baseline interview and health examination between 2001 and 2002. Enrolled participants (*n* = 5018) visited the study site for biennial follow-up interviews and health examinations. The most recent follow-up was completed between 2023 and 2024, with datasets from 2001 to 2020 currently available.

Serum vitamin B12, folate, and Hcy levels were exclusively measured among 2827 participants between 2007 and 2008 (among 3433 participants followed up, serum samples from 606 participants were insufficient for assays), establishing this period as the baseline for the current study. At baseline, muscle mass percentage (MMP) was estimated using bioelectrical impedance analysis, rather than HS measurement, to identify individuals with PS. A previous report defined sarcopenia as MMP < 37% for men and <27.6% for women [18]. In the current study, the MMP of all participants, excluding those with missing data (*n* = 20), exceeded 44%. HS was measured between 2013 and 2014, designated as the endpoint period. Among 2807 participants (ages: 45–76 years) with available baseline data on biomarkers and MMP, individuals with anemia defined as Hb levels < 13 g/dL in men or <12 g/dL in women (*n* = 386), those with a cancer diagnosis (*n* = 17), and those exhibiting potential infection or inflammation defined by white blood cell (WBC) counts >11,000 cells/μL or high-sensitivity C-reactive protein (hs-CRP) levels > 5 mg/L (*n* = 178) were excluded. Among remaining 2226 participants, because 296 were lost to follow-up (a major reason for non-participation was lack of time) for HS measurement, 1930 participants without PS at baseline (follow-up rate: 86.7%) were included in this study (Supplementary Materials).

The KNIH obtained written informed consent from all participants of the cohort study and released datasets after all personally identifiable information was removed. All methods of the cohort study were conducted in accordance with the Declaration of Helsinki and local ethical guidelines and regulations concerning human research. The current study was approved by the Institutional Review Board of Kookmin University (KMU-202312-HR-384) with permission from the KNIH.

### 2.2. HS Measurement and PS as an Outcome

HS was measured by trained researchers utilizing a hydraulic grip dynamometer. Following the method similarly used in a previous study [19], participants were seated with their second and fourth fingers positioned at the mid-joints and their elbows bent at a 90° angle. Maximum strength was assessed three times for 3 s in each hand, and the average of six measurements (three from each hand) was calculated to determine each individual’s absolute HS value.

The outcome of this study was PS defined as absolute HS values < 28 kg for men and <18 kg for women according to the criteria for Asians [3].

### 2.3. Assay of Serum Biomarkers

Participants’ blood samples were collected after an overnight fast. The exposures of this study included serum vitamin B12, folate, Hcy, and ferritin levels, which were assessed using a chemiluminescent immunoassay method. Serum aspartate transaminase (AST), uric acid (UA), creatinine (Cr), and hs-CRP levels, as well as a complete blood count test, which included WBC count and Hb, were assessed utilizing automated analyzers (Siemens, Tarrytown, NY, USA). These biomarkers were analyzed in a commercial laboratory, which conducted routine quality control assessments.

### 2.4. Confounding Variables

On the basis of previous reports [5,20], demographic and socioeconomic characteristics, lifestyle factors, prevalent chronic diseases, serum biomarkers, and anthropometric measures were considered confounding variables in this study. Demographic and socioeconomic characteristics including age, sex, monthly income, education level, and occupation, as well as disease history and lifestyle factors such as smoking status, alcohol consumption, physical activity, and dietary protein intake, were collected through questionnaire-based interviews conducted by trained researchers. The lifestyle questionnaire has been detailed in a previous report [17]. Average daily protein intake was estimated using a food frequency questionnaire (FFQ), which was developed and validated by the Korea Disease Control and Prevention Agency (Seoul, Republic of Korea) [17], and the food composition database published by the Rural Development Administration of Korea [21]. The FFQ asks for information about the average consumption frequency (categories: “almost never,” “once a month,” “2–3 times a month,” “1–2 times a week,” “3–4 times a week,” “5–6 times a week,” “once a day,” “twice a day,” or “3 times a day”) and serving size (categories: “larger than,” “equal to,” or “smaller than” a standard serving size) of 106 food items and beverages consumed in the previous year. To calculate the daily average consumption frequency of each food item, the frequency was multiplied by 1.5 for larger amounts, 1 for an equal amount, and 0.5 for smaller amounts as compared to the standard serving size. Although the questionnaire includes a question regarding supplement intake, it does not include a question about supplements for specific nutrients, so protein supplement intake was not considered to calculate average daily protein intake. Additional questionnaire data concerning the diagnosis, treatment, and medication for diabetes mellitus (DM), hypertension (HTN), and cardiovascular disease (CVD) were collected. To identify the prevalence of DM and HTN at baseline, on-site measurements of serum glucose levels and blood pressure were performed. Furthermore, on-site anthropometric measurements were conducted to obtain body mass index (BMI), calculated by dividing body weight (kg) by the square of the height (m), and MMP, estimated via bioelectrical impedance analysis (Biospace Co., Ltd., Seoul, Republic of Korea).

### 2.5. Statistical Analysis

Descriptive statistics were generated according to sex-specific quartiles of absolute HS, presented as the mean ± standard deviation or percentage. Statistical differences across quartiles were assessed using the chi-square test and analysis of variance (ANOVA) for categorical and continuous variables, respectively. *p*-values for linear trends were determined using the Cochran-Armitage and ANOVA trend tests.

To examine the associations between serum biomarkers, as independent variables, and PS, as a dependent binary variable, modified Poisson regression analysis was employed to derive risk ratios (RRs) and 95% confidence intervals (CIs) for each biomarker. The biomarkers were analyzed both as quartiles and as categorical variables [22,23]. Serum vitamin B12 levels were categorized as follows: ≤300 pg/mL for low or deficient levels, 301–500 pg/mL for low-normal levels, 501–700 pg/mL for moderate-normal levels, and >700 pg/mL for high-normal levels. Serum folate levels were categorized as follows: ≤4 ng/mL for low or deficient levels, 4.1–7 ng/mL for low-normal levels, 7.1–10 ng/mL for moderate-normal levels, and >10 ng/mL for high-normal levels. Serum Hcy levels were categorized as follows: ≤9 μmol/L for low-normal levels, 9.1–12 μmol/L for moderate-normal levels, 12.1–15 μmol/L for high-normal levels, and >15 μmol/L for elevated levels. Serum ferritin levels were categorized as follows: ≤50 ng/mL for low or deficient levels, 51–100 ng/mL for low-normal levels, 101–200 ng/mL for high-normal levels, 201–300 ng/mL for mildly elevated levels, and >300 ng/mL for elevated levels.

In the multiple regression models for each independent variable, potential confounding factors included age (continuous), sex, BMI (continuous), muscle mass percentage (continuous), income level (≤2 × 10^6^ or >2 × 10^6^ won monthly, categorized based on data for families of four receiving livelihood benefits [https://www.mohw.go.kr]), educational level (≤middle school or ≥high school), occupation (office worker or other (including missing data)), smoking status (four categories: non-smoker, former smoker or non-responder, current smoker: ≤10 cigarettes/day and >10 cigarettes/day), alcohol consumption (five categories: non-drinker, former drinker or non-responder, current drinker: alcohol consumption ≤ 15 g/day, 15.1–30 g/day, and >30 g/day), weekly exercise frequency (three categories: <1 day, 1–4 days, ≥5 days), average daily protein intake (quartiles), menopausal status for women (yes or no), presence of DM, HTN, or CVD (yes or no), and continuous blood biomarkers including WBC counts, Hb, AST, UA, Cr, and hs-CRP. Interactions between exposures and confounding variables known to influence sarcopenia, such as age, muscle mass, physical activity, and protein intake, were further evaluated by adding an interaction term in the model. Data for confounding variables, except protein intake, were sourced from the 2007–2008 follow-up dataset (the baseline dataset for the current study). Protein intake data were obtained from the 2005–2006 follow-up dataset, as the FFQ was not utilized between 2007 and 2014. All analyses were conducted using the SAS program (SAS 9.4, SAS Institute, Cary, NC, USA) with a two-sided significance level set at *p* < 0.05.

## 3. Results

### 3.1. Baseline Characteristics by HS Quartiles

Among the 1930 study participants (1060 women and 870 men), 598 PS cases were identified over the 6-year study period. Baseline characteristics across the sex-specific quartiles of absolute HS are summarized in Table 1, which includes *p*-values for trend. Participants with higher absolute HS values were generally younger, heavier, and more muscular, and they hailed from higher income and educational backgrounds. They were predominantly office workers, current smokers, current alcohol consumers, physically active, and free from chronic diseases. Furthermore, these individuals exhibited higher protein intake, alongside elevated serum levels of vitamin B12, Hb, and UA, while exhibiting lower WBC counts and serum Hcy, AST, and hs-CRP levels.

### 3.2. Associations of Serum Vitamin B12, Folate, Hcy, and Ferritin Quartiles with PS Risk

Table 2 presents the associations between serum biomarkers and PS, defined as low HS assessed at 6-year follow-up. In multivariable models adjusted for potential confounders, an inverse trend was noted for serum vitamin B12 in relation to PS risk (*p* for trend = 0.06), although no significant associations were observed for individual quartiles. Participants in the second, third, and fourth quartiles of serum Hcy and the third quartile of serum ferritin demonstrated significantly lower risks. The multivariable RRs (95% CI) for the lowest risk were 0.74 (0.62, 0.88) for the second quartile of Hcy and 0.81 (0.68, 0.95) for the third quartile of ferritin compared with the lowest quartile of each biomarker. Additionally, no association was examined between serum folate levels and PS risk.

### 3.3. Associations of Serum Vitamin B12, Folate, Hcy, and Ferritin Categories with PS Risk in All, Male, and Female Participants

Table 3 details the sex-specific associations between serum biomarker categories and PS risk adjusted for confounding variables. For serum Hcy, a significant decrease in PS risk was observed in the moderate-normal (9.1–12 μmol/L), high-normal (12.1–15 μmol/L), and elevated (>15 μmol/L) categories among men, while a borderline significant reduction was noted in the high-normal category among women. Compared with the low-normal category, the multivariable RRs (95% CI) for PS associated with Hcy were 0.59 (0.38, 0.90), 0.61 (0.39, 0.97), and 0.61 (0.38, 0.99) for the moderate- to high-normal categories among men, and 0.80 (0.64, 1.00) for the high-normal category among women. Nevertheless, vitamin B12 and folate categories were not significantly associated with PS risk in either sex. Additionally, individuals in the high-normal category of serum ferritin (101–200 ng/mL) exhibited a significant reduction in PS risk, both for men and women, as well as across the entire cohort. Compared with the low or deficient category, the multivariable RRs (95% CI) associated with the high-normal category were 0.70 (0.50, 0.97) for men and 0.79 (0.66, 0.95) for women.

### 3.4. Joint Associations of Serum Hcy and Ferritin Levels and Risk Factors with PS Risk

Table 4 presents the results for interactions among Hcy, ferritin, and potential baseline risk factors, including age, muscle mass, physical activity, and protein intake, in relation to PS risk. In the analysis encompassing all participants, significant risk factors for sarcopenia included age ≥ 60 years, muscle mass < 70%, and the absence of exercise. In joint analysis of age and elevated Hcy levels defined as >15 μmol/L, the multivariable RRs (95% CI) were 0.23 (0.06, 0.90) for participants under 60 years and 3.49 (2.71, 4.50) for those aged ≥60 years compared with those aged under 60 years with Hcy levels ≤ 15 μmol/L. Furthermore, a significant interaction between age and elevated Hcy was identified (P for interaction < 0.05). Conversely, no significant interactions were found between other risk factors and elevated Hcy. However, no significant interactions were noted in joint analyses for risk factors and elevated ferritin levels defined as >300 ng/mL for men and >200 ng/mL for women.

**Figure 1:**
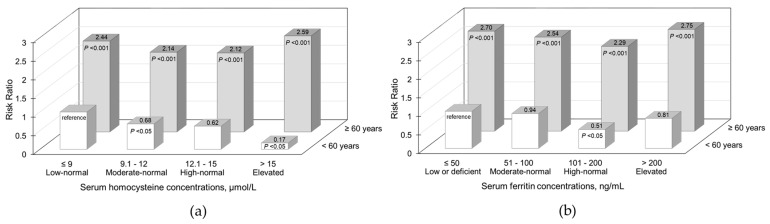
Associations of serum homocysteine and ferritin levels and probable sarcopenia risk stratified by age groups: The results are illustrated for serum homocysteine (**a**) and serum ferritin (**b**). Risk ratios (Y axis) of probable sarcopenia for serum homocysteine and ferritin categories (X axis) were estimated using references, adults younger than 60 years with low-normal homocysteine levels (**a**) and those with low or deficient ferritin levels (**b**), after data are adjusted for age (continuous), sex, body mass index (continuous), muscle mass percentage (continuous), income level (≤2 × 10^6^ or >2 × 10^6^ won monthly), educational level (≤middle school or ≥high school), occupational type (office worker or other), smoking status (four categories: non-smoker, former smoker or non-responder, current smoker: ≤10 cigarettes/day and >10 cigarettes/day), alcohol consumption (five categories: non-drinker, former drinker or non-responder, current drinker: alcohol consumption ≤ 15 g/day, 15.1–30 g/day, and >30 g/day), weekly exercise frequency (three categories: <1 day, 1–4 days, ≥5 days), average daily protein intake (quartiles), presence of diabetes mellitus, hypertension, or cardiovascular disease (yes or no), and blood biomarkers (continuous) including white blood cell counts, hemoglobin, aspartate aminotransferase, uric acid, creatinine, and high-sensitivity C-reactive protein, vitamin B12, folate, homocysteine, and ferritin.

Further analysis results stratified by age groups (<60 and ≥60 years) are illustrated in Figure 1, in which the RRs for PS were estimated using reference categories, such as adults younger than 60 years with low-normal Hcy levels and those with low or deficient ferritin levels. Elevated Hcy increased PS risk exclusively in older individuals (Figure 1a), while elevated ferritin increased the risk in both younger and older participants (Figure 1b). In younger participants, elevated Hcy was associated with a decrease in PS risk; the RRs (95% CI) for PS were 0.68 (0.46, 0.99) for the moderate-normal category, 0.62 (0.36, 1.06) for the high-normal category, and 0.17 (0.05, 0.67) for the elevated category, compared with the low-normal category (Figure 1a). Conversely, among older individuals, the RRs (95% CI) for PS associated with Hcy were 1.15 (0.95, 1.40) for the low-normal category, 1.01 (0.86, 1.19) for the moderate-normal category, and 1.22 (1.00, 1.48) for the elevated category, compared with the high-normal category (data available upon request).

## 4. Discussion

This 6-year prospective study investigated the associations of serum vitamin B12, folate, Hcy, and ferritin levels with PS risk defined as low HS among middle-aged and older adults without anemia at baseline. Among all participants, PS risk was lowest in the high-normal ranges of serum Hcy (12.1–15 μmol/L) and ferritin (101–200 ng/mL) levels, and such a reduction in the risk was not observed in the low-normal or elevated ranges. Similar associations for ferritin were observed in both sex- and age-specific contexts. A comparable association was noted for Hcy in sex-specific analyses whereas the associations varied according to age group, indicating that age was a significant modifier. Notably, elevated Hcy decreased PS risk in middle-aged participants, while it increased the risk among older adults.

Sarcopenia is a progressive, age-related condition characterized by the loss of muscle mass, strength, and function, leading to adverse geriatric outcomes such as falls, fractures, disability, frailty, and mortality. Its estimated prevalence in the older population worldwide is approximately 10% and is expected to rise rapidly as the number of older individuals increases [24]. While several risk factors for sarcopenia have been identified, with age being the most significant, data on various modifiable factors that potentially play a crucial role in the prevention and management of sarcopenia remain limited [4].

Recent studies have underlined the potential role of vitamins and minerals, such as vitamin B12, folate, and iron in preventing sarcopenia [25]. An accumulating body of clinical and epidemiological evidence supports a connection between anemia—diagnosed primarily based on Hb levels—and sarcopenia [12]. Nevertheless, the coexistence of these two conditions and their mutual influence, along with the fact that low Hb can result from both iron deficiency and deficiencies in vitamin B12 and folate, complicates the establishment of a causal relationship between each of these nutrients and sarcopenia.

Serum or plasma ferritin is regarded as a considerably sensitive and specific biomarker for assessing iron status, detecting iron deficiency earlier than Hb levels [26]. Ferritin serves as the primary protein for iron storage within body tissues, mostly in the liver, and its blood levels are also correlated with skeletal muscle ferritin content [27]. Because ferritin plays a dual role as an iron-status marker and an acute-phase reactant, however, its levels may be elevated in the context of acute inflammation, which can obscure true iron deficiency [26].

In the present study, individuals with elevated WBC counts and hs-CRP levels—markers indicative of potential inflammation or infection— were excluded at baseline and these markers were considered as confounding factors. After further adjustment for serum vitamin B12 and folate levels, a significant association between high-normal ferritin levels and PS risk was observed. Contrary to previous studies [14,20], however, this study did not examine whether elevated ferritin levels increase PS risk.

Hcy is produced as an intermediate byproduct of methionine metabolism, wherein methionine synthase catalyzes the re-methylation of Hcy to methionine by transferring a methyl group from methyltetrahydrofolate with the cofactor vitamin B12, producing methionine and tetrahydrofolate. Additionally, Hcy undergoes irreversible conversion into cysteine, a precursor for glutathione, via the transsulfuration (TS) pathway that involves the cofactor vitamin B6. Consequently, deficiencies in vitamins B12, B9 (folate), and B6 potentially impair methionine and cysteine production, leading to Hcy accumulation. Excessive Hcy accumulation can heighten oxidative stress [28], which is considered a potential biological mechanism underlying aging-related muscle weakness [29].

In the present study, elevated Hcy levels were found to be associated with increased PS risk in older participants, in contrast to their association with decreased risk in middle-aged participants. Furthermore, among individuals with elevated Hcy levels, older participants were observed to have significantly lower serum vitamin B12 levels than middle-aged participants (data available upon request). These results may suggest that older participants are more likely to have disrupted methionine metabolism associated with lowered vitamin B12 levels. Consequently, they might have higher levels of oxidative stress due to aging and Hcy accumulation compared to middle-aged adults. On the other hand, elevated Hcy levels of middle-aged participants might be due to other factors, such as high intake of methionine, which is critical for muscle protein synthesis and promoting muscle growth [30]. Further investigations are warranted to clarify the biological mechanisms underlying the age-specific associations between Hcy levels and PS risk.

The strengths of this study include the utilization of population-based prospective cohort data and a comprehensive range of variables. Notwithstanding, several study limitations must be considered when interpreting the results. At baseline, HS was not measured; instead, MMP was assessed as an alternative measure to exclude prevalent cases and was considered a confounder. HS-based PS cases might not be excluded completely at baseline although the number of these cases might be trivial because all participants aged 45–76 years showed baseline MMP over 44%, which is generally considered high. Moreover, serum levels of vitamin B6, a cofactor in the TS pathway, were not evaluated. Although random errors might occur in the biomarkers and HS assessments, potentially leading to a failure to find associations, trained researchers performed these assessments to minimize errors. Other study limitations are the measurement random errors regarding protein intake due to the use of FFQ and the temporal mismatch between dietary intake and biomarkers, as a source of residual confounding. Furthermore, the applicability of the findings may be limited to adults within comparable age ranges and ethnicities.

## 5. Conclusions

In summary, serum Hcy levels between 9 and 15 μmol/L, as well as normal serum ferritin levels between 100 and 200 ng/mL, were associated with the lowest PS risk in a 6-year prospective study involving middle-aged and older adults. Elevated levels of Hcy or ferritin above the normal range potentially contribute to age-related muscle weakness. Therefore, maintaining muscle strength in middle-aged and older adults necessitates strategies aimed at achieving optimal Hcy and ferritin levels, including the prevention of vitamin B deficiencies.

## Figures and Tables

**Table 1:** Baseline characteristics of 1930 study participants across the sex-specific quartiles of handgrip strength assessed at the 6-year follow-up.

Characteristics	Sex-Specific Quartiles of Absolute Handgrip Strength				*p* Value for Trend ^1^
	1st Quartile	2nd Quartile	3rd Quartile	4th Quartile	
Number of participants	482	488	485	475	
Relative handgrip strength	0.77 ± 0.30	1.0 ± 0.4	1.2 ± 0.40	1.4 ± 0.4	<0.001
Age, years	66.1 ± 7.2	62.0 ± 7.6	57.6 ± 7.0	53.7 ± 5.8	<0.001
Body mass index, kg/m^2^	24.3 ± 3.3	24.3 ± 3.0	25.0 ± 3.1	25.1 ± 3.1	<0.001
Muscle mass percentage	68.8 ± 7.3	69.8 ± 7.2	69.8 ± 7.1	70.8 ± 6.9	<0.001
Low income ^2^, %	87.3	79.7	69.1	57.3	<0.001
High school or higher, %	14.7	21.1	27.8	40.4	<0.001
Office worker, %	1.2	3.7	6.6	7.0	<0.001
Current smoker, %	16.2	15.8	16.9	20.4	0.07
Current alcohol drinker, %	40.7	40.6	46.8	53.3	<0.001
Regular exercise ≥ 5 days/week, %	9.8	8.8	13.4	12.8	<0.05
Presence of chronic diseases ^3^, %	52.3	50.0	46.8	43.0	<0.01
Dietary protein intake, g/day	52.9 ± 26.6	55.9 ± 34.0	58.4 ± 37.3	60.4 ± 34.6	<0.001
Blood biomarkers					
Vitamin B12, pg/mL	573.0 ± 339.0	601.5 ± 248.9	628.4 ± 334.2	625.6 ± 283.8	<0.01
Folate, ng/mL	7.3 ± 4.1	7.6 ± 4.8	7.7 ± 4.4	7.7 ± 4.2	0.23
Homocysteine, μmol/L	12.4 ± 4.4	11.6 ± 3.2	11.2 ± 3.9	11.1 ± 4.7	<0.001
Ferritin, ng/mL	139.8 ± 137.2	130.9 ± 117.6	141.8 ± 141.4	136.6 ± 125.4	0.96
White blood cell count, thousand/μL	6.3 ± 1.7	6.0 ± 1.5	6.2 ± 1.7	6.0 ± 1.7	<0.05
Hemoglobin, g/dL	13.8 ± 1.1	13.9 ± 1.1	14.0 ± 1.3	14.0 ± 1.2	<0.05
Aspartate aminotransferase, IU/L	26.6 ± 11.1	26.5 ± 10.3	25.5 ± 8.8	25.3 ± 9.6	<0.05
Uric acid, mg/dL	4.8 ± 1.4	4.7 ± 1.4	4.9 ± 1.3	4.9 ± 1.3	<0.05
Creatinine, mg/dL	0.91 ± 0.15	0.91 ± 0.14	0.91 ± 0.13	0.92 ± 0.14	0.75
Hs-CRP, mg/L	1.1 ± 1.0	0.94 ± 0.83	1.1 ± 0.94	0.89 ± 0.83	<0.05

Abbreviation: Hs-CRP, high-sensitivity C-reactive protein. Mean ± standard deviation or proportions in the cell. ^1^ Obtained using the Cochran-Armitage and analysis of variance trend tests. ^2^ Average monthly wage < 2 × 10^6^ won. ^3^ Presence of diabetes mellitus, hypertension, or cardiovascular disease.

**Table 2:** Associations of serum vitamin B12, folate, homocysteine, and ferritin quartiles with probable sarcopenia risk in all participants.

Serum Biomarkers	Quintiles (Q)	Cases	Risk Ratio (95% Confidence Interval)		
	[Median]		Age-Adjusted	Multivariable Model 1	Multivariable Model 2
Vitamin B12, pg/mL	1st Q [375]	185	Reference	Reference	Reference
	2nd Q [499]	149	0.97 (0.83, 1.14)	0.93 (0.79, 1.09)	0.94 (0.80, 1.10)
	3rd Q [616]	129	0.95 (0.81, 1.12)	0.87 (0.74, 1.03)	0.88 (0.74, 1.03)
	4th Q [832]	135	0.98 (0.83, 1.15)	0.87 (0.74, 1.02)	0.86 (0.73, 1.01)
	*p* for trend		0.79	0.07	0.06
Folate, ng/mL	1st Q [3.6]	131	Reference	Reference	Reference
	2nd Q [5.6]	165	1.18 (0.99, 1.39)	1.07 (0.90, 1.27)	1.07 (0.90, 1.27)
	3rd Q [7.7]	160	1.19 (1.00, 1.41)	1.04 (0.88, 1.24)	1.06 (0.89, 1.26)
	4th Q [12.1]	142	1.14 (0.95, 1.36)	0.98 (0.82, 1.18)	0.97 (0.80, 1.18)
	*p* for trend		0.29	0.62	0.55
Homocysteine, μmol/L	1st Q [8.1]	150	Reference	Reference	Reference
	2nd Q [10.1]	131	0.70 (0.59, 0.83)	0.75 (0.63, 0.89)	0.74 (0.62, 0.88)
	3rd Q [11.9]	149	0.69 (0.58, 0.82)	0.79 (0.67, 0.94)	0.77 (0.64, 0.92)
	4th Q [15.1]	168	0.67 (0.57, 0.79)	0.86 (0.72, 1.03)	0.81 (0.67, 0.98)
	*p* for trend		<0.001	0.36	0.15
Ferritin, ng/mL	1st Q [44]	174	Reference	Reference	Reference
	2nd Q [86]	166	0.83 (0.72, 0.97)	0.95 (0.82, 1.11)	0.98 (0.84, 1.14)
	3rd Q [132]	133	0.65 (0.55, 0.76)	0.79 (0.67, 0.93)	0.81 (0.68, 0.95)
	4th Q [232]	125	0.66 (0.56, 0.79)	0.92 (0.76, 1.10)	0.95 (0.78, 1.15)
	*p* for trend		<0.001	0.23	0.28

In multivariable model 1, data are adjusted for age (continuous), sex, body mass index (continuous), muscle mass percentage (continuous), income level (≤2 × 10^6^ or >2 × 10^6^ won monthly), educational level (≤middle school or ≥high school), occupational type (office worker or other), smoking status (four categories: non-smoker, former smoker or non-responder, current smoker: ≤10 cigarettes/day and >10 cigarettes/day), alcohol consumption (five categories: non-drinker, former drinker or non-responder, current drinker: alcohol consumption ≤ 15 g/day, 15.1–30 g/day, and >30 g/day), weekly exercise frequency (three categories: <1 day, 1–4 days, ≥5 days), average daily protein intake (quartiles), presence of diabetes mellitus, hypertension, or cardiovascular disease (yes or no), and blood biomarkers (continuous) including white blood cell counts, hemoglobin, aspartate aminotransferase, uric acid, creatinine, and high-sensitivity C-reactive protein. In multivariable model 2, data are adjusted for serum levels of vitamin B12, folate, homocysteine, and ferritin with the covariates of model 1.

**Table 3:** Associations of serum vitamin B12, folate, homocysteine, and ferritin categories with probable sarcopenia risk in all, male, and female participants.

Serum Biomarkers	Categories	Cases	Multivariable Risk Ratio (95% Confidence Interval)		
	[Range]	Noncases	All	Men, *n* = 870	Women, *n* = 1060
Vitamin B12, pg/mL	Low or deficient [≤300]	11/32	Reference	Reference	Reference
	Low-normal [301–500]	97/125	0.95 (0.78, 1,16)	1.23 (0.79, 1.90)	0.86 (0.69, 1.07)
	Moderate-normal [501–700]	49/152	0.87 (0.71, 1.07)	0.87 (0.53, 1.42)	0.91 (0.73, 1.13)
	High-normal [>700]	26/106	0.84 (0.67, 1.04)	0.75 (0.43, 1.30)	0.89 (0.70, 1.14)
Folate, ng/mL	Low or deficient [≤4]	53/42	Reference	Reference	Reference
	Low-normal [4.1–7.0]	74/159	0.97 (0.82, 1.16)	1.09 (0.82, 1.44)	0.96 (0.77, 1.21)
	Moderate-normal [7.1–10]	34/119	0.98 (0.81, 1.19)	0.95 (0.65, 1.39)	1.00 (0.79, 1.27)
	High-normal [>10]	22/95	0.90 (0.72, 1.11)	0.93 (0.61, 1.42)	0.89 (0.69, 1.16)
Homocysteine, μmol/L	Low-normal [≤9]	18/121	Reference	Reference	Reference
	Moderate-normal [9.1–12]	56/172	0.77 (0.65, 0.90)	0.59 (0.38, 0.90)	0.85 (0.72, 1.02)
	High-normal [12.1–15]	54/88	0.73 (0.60, 0.89)	0.61 (0.39, 0.97)	0.80 (0.64, 1.00)
	Elevated [>15]	55/34	0.82 (0.66, 1.02)	0.61 (0.38, 0.99)	0.88 (0.67, 1.14)
Ferritin, ng/mL	Low or deficient [≤50]	18/107	Reference	Reference	Reference
	Low-normal [51–100]	37/160	0.89 (0.76, 1.04)	0.86 (0.59, 1.25)	0.89 (0.75, 1.05)
	High-normal [101–200]	63/122	0.75 (0.64, 0.87)	0.70 (0.50, 0.97)	0.79 (0.66, 0.95)
	Mildly elevated [201–300]	38/20	0.91 (0.71, 1.17)	0.94 (0.66, 1.34)	0.90 (0.62, 1.31)
	Elevated [>300]	27/6	0.86 (0.64, 1.16)	0.83 (0.55, 1.25)	1.03 (0.63, 1.69)

In multivariable model 1, data are adjusted for age (continuous), sex, body mass index (continuous), muscle mass percentage (continuous), income level (≤2 × 10^6^ or >2 × 10^6^ won monthly), educational level (≤middle school or ≥high school), occupational type (office worker or other), smoking status (four categories: non-smoker, former smoker or non-responder, current smoker: ≤10 cigarettes/day and >10 cigarettes/day), alcohol consumption (five categories: non-drinker, former drinker or non-responder, current drinker: alcohol consumption ≤ 15 g/day, 15.1–30 g/day, and >30 g/day), weekly exercise frequency (three categories: <1 day, 1–4 days, ≥5 days), average daily protein intake (quartiles), menopausal status for women (yes or no), presence of diabetes mellitus, hypertension, or cardiovascular disease (yes or no), and blood biomarkers (continuous) including white blood cell counts, hemoglobin, aspartate aminotransferase, uric acid, creatinine, and high-sensitivity C-reactive protein, vitamin B12, folate, homocysteine, and ferritin.

**Table 4:** Joint associations between elevated serum levels of serum ferritin and homocysteine and potential risk factors with probable sarcopenia risk.

Risk Factors at Baseline	Cases	RR ^1^ (95% CI)	RR ^1^ (95% CI) by Hcy Levels		RR ^1^ (95% CI) by Ferritin Levels ^2^	
	Noncases	All	≤15 μmol/L	>15 μmol/L	≤200 or 300 ng/mL	>200 or 300 ng/mL
Age						
<60 years	110/835	Reference	Reference	0.23 (0.06, 0.90)	Reference	1.18 (0.64, 2.17)
≥60 years	488/497	3.09 (2.52, 3.78)	2.81 (2.29, 3.44)	3.49 (2.71, 4.50)	3.08 (2.50, 3.81)	3.14 (2.31, 4.26)
*p* for interaction			<0.05	0.66		
Muscle mass						
≥70%	198/728	Reference	Reference	1.14 (0.88, 1.46)	Reference	1.03 (0.75, 1.41)
<70%	400/604	1.44 (1.17, 1.78)	1.48 (1.19, 1.83)	1.63 (1.23, 2.15)	1.45 (1.17, 1.79)	1.55 (1.09, 2.19)
*p* for interaction			0.85	0.86		
Physical activity						
Regular exercise	126/469	Reference	Reference	1.05 (0.71, 1.55)	Reference	1.29 (0.73, 2.28)
Absence of exercise	472/863	1.19 (1.02, 1.39)	1.18 (0.99, 1.40)	1.30 (1.03, 1.64)	1.22 (1.03, 1.43)	1.17 (0.91, 1.52)
*p* for interaction			0.81	0.35		
Protein intake						
2nd to 4th quartiles	427/1038	Reference	Reference	1.05 (0.85, 1.30)	Reference	0.93 (0.71, 1.22)
1st quartile	171/294	0.96 (0.84, 1.10)	0.95 (0.82, 1.09)	1.10 (0.86, 1.39)	0.94 (0.82, 1.07)	1.23 (0.87, 1.73)
*p* for interaction			0.46	0.13		

Abbreviation: RR, risk ratio; CI, confidence interval; Hcy, homocysteine. ^1^ For produce multivariable risk ratios, data are adjusted for age (continuous), sex, body mass index (continuous), muscle mass percentage (continuous), income level (≤2 × 10^6^ or >2 × 10^6^ won monthly), educational level (≤middle school or ≥high school), occupational type (office worker or other), smoking status (four categories: non-smoker, former smoker or non-responder, current smoker: ≤10 cigarettes/day and >10 cigarettes/day), alcohol consumption (five categories: non-drinker, former drinker or non-responder, current drinker: alcohol consumption ≤ 15 g/day, 15.1–30 g/day, and >30 g/day), weekly exercise frequency (three categories: <1 day, 1–4 days, ≥5 days), average daily protein intake (quartiles), presence of diabetes mellitus, hypertension, or cardiovascular disease (yes or no), and blood biomarkers (continuous) including white blood cell counts, hemoglobin, aspartate aminotransferase, uric acid, creatinine, and high-sensitivity C-reactive protein, vitamin B12, folate, homocysteine, and ferritin. ^2^ Elevated ferritin levels indicating overload status are defined as >300 ng/mL for men and >200 ng/mL for women.

## Data Availability

Raw data are available through the National Biobank of Korea, following the data distribution procedure described on its website (https://biobank.nih.go.kr/eng, accessed on 27 March 2026).

## Supplementary Materials

The following supporting information can be downloaded at: https://www.mdpi.com/article/10.3390/nu18091362/s1. Flow diagram for participants in the study.

## Abbreviations

The following abbreviations are used in this manuscript:

ANOVA	analysis of variance
AST	aspartate transaminase
BMI	body mass index
CI	confidence interval
Cr	creatinine
CVD	cardiovascular disease
DM	diabetes mellitus
FFQ	food frequency questionnaire
HS	handgrip strength
Hb	hemoglobin
Hcy	homocysteine
hs-CRP	high-sensitivity C-reactive protein
HTN	hypertension
KNIH	Korea National Institute of Health
KoGES	Korean Genome and Epidemiology Study
MMP	muscle mass percentage
RR	risk ratio
SD	standard deviation
PS	probable sarcopenia
TS	transsulfuration
UA	uric acid
WBC	white blood cell
