# Supplementary figures and images for: Prospective Associations of Serum Vitamin B12, Homocysteine, and Ferritin Levels with Probable Sarcopenia
Source: Nutrients. 2026 Apr 25;18(9):1362. doi: 10.3390/nu18091362 (PMC13164896; doi:10.3390/nu18091362)

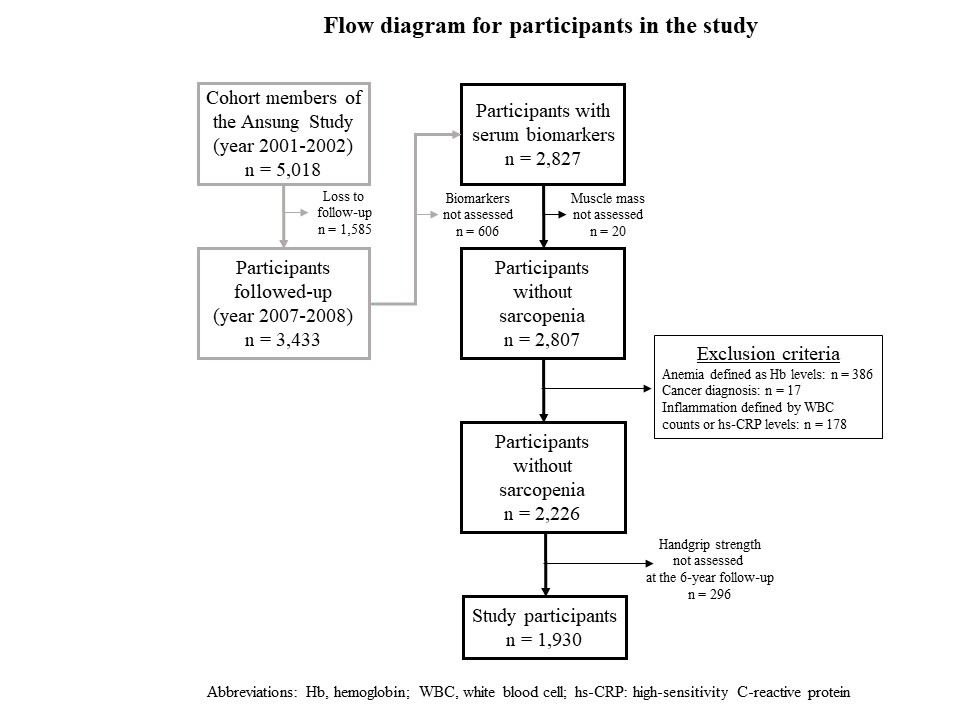

Supplement: Supplementary file 1 [file nutrients-18-01362-s001.zip › nutrients-4253460-supplementary.jpg]
